# OncoEducate: a pilot study of generative AI to enhance patient–clinician communication in genitourinary cancer care

**DOI:** 10.1093/oncolo/oyag135

**Published:** 2026-04-09

**Authors:** Henry K Litt, Amelia Wodzinski, Pearl Subramanian, Mackenzie Donovan, Neha Vapiwala, Vivek Narayan, Lin Mei, Samuel Takvorian, Naomi B Haas, Ravi B Parikh, Ronak Mistry, Ronac Mamtani

**Affiliations:** Division of Hematology/Oncology, Department of Medicine, University of Pennsylvania, Philadelphia, PA, 19104, United States; Abramson Cancer Center, University of Pennsylvania, Philadelphia, PA, 19104, United States; Abramson Cancer Center, University of Pennsylvania, Philadelphia, PA, 19104, United States; Department of Medicine, University of Pennsylvania, Philadelphia, PA, 19104, United States; Abramson Cancer Center, University of Pennsylvania, Philadelphia, PA, 19104, United States; Abramson Cancer Center, University of Pennsylvania, Philadelphia, PA, 19104, United States; Department of Radiation Oncology, University of Pennsylvania, Philadelphia, PA, 19104, United States; Division of Hematology/Oncology, Department of Medicine, University of Pennsylvania, Philadelphia, PA, 19104, United States; Abramson Cancer Center, University of Pennsylvania, Philadelphia, PA, 19104, United States; Division of Hematology/Oncology, Department of Medicine, University of Pennsylvania, Philadelphia, PA, 19104, United States; Abramson Cancer Center, University of Pennsylvania, Philadelphia, PA, 19104, United States; Division of Hematology/Oncology, Department of Medicine, University of Pennsylvania, Philadelphia, PA, 19104, United States; Abramson Cancer Center, University of Pennsylvania, Philadelphia, PA, 19104, United States; Division of Hematology/Oncology, Department of Medicine, University of Pennsylvania, Philadelphia, PA, 19104, United States; Abramson Cancer Center, University of Pennsylvania, Philadelphia, PA, 19104, United States; Department of Hematology and Medical Oncology, Emory University School of Medicine, Atlanta, GA, 30322, United States; Division of Hematology/Oncology, Department of Medicine, University of Pennsylvania, Philadelphia, PA, 19104, United States; Division of Hematology/Oncology, Department of Medicine, University of Pennsylvania, Philadelphia, PA, 19104, United States; Abramson Cancer Center, University of Pennsylvania, Philadelphia, PA, 19104, United States

**Keywords:** artificial intelligence, patient education, genitourinary oncology, treatment intent

## Abstract

OncoEducate is a clinician-supervised generative artificial intelligence (AI) application designed to deliver standardized, regimen-level, plain-language education at the point of care. We developed a templated handout generator using a locked prompt and predefined content structure that incorporates diagnosis, treatment regimen, and treatment intent, with clinician review prior to patient distribution. We then conducted a prospective, two-phase pilot at a single academic cancer center. In Phase I, six clinicians evaluated AI-generated handouts for nine commonly used palliative-intent genitourinary (GU) oncology regimens using 7-point Likert-scale items. In Phase II, patients with advanced kidney, prostate, or urothelial cancers initiating palliative-intent therapy received clinician-reviewed handouts and completed follow-up surveys at the next office or infusion visit or by telephone when in-person completion was not feasible. Clinicians rated handouts as useful (median 6/7) and accessible (median 6.5/7), with median accuracy ratings of 6-7/7 across domains. During an 8-week period, 20 of 21 approached patients enrolled (95%). Patients rated handouts as informative and readable (median 7/7 for both), and 65% correctly identified treatment intent as palliative. These findings support the feasibility and acceptability of clinician-reviewed, templated AI-generated handouts in routine GU oncology care, justifying larger randomized studies to evaluate clinical impact.

## Introduction

Patients with cancer receive complex information about their care, leading to misunderstandings that contribute to discordant decision-making and low-value end-of-life care.^1–3^ Up to 80% of patients with advanced solid tumors believe treatment may cure them.^3^ Existing educational strategies are difficult to scale.^4^^,^^5^

Generative artificial intelligence (AI) may help translate medical information into plain-language explanations at scale.^6^^,^^7^ We developed OncoEducate, a clinician-supervised, templated application built on a large language model (LLM) that generates diagnosis- and regimen-tailored handouts emphasizing treatment intent. We conducted a prospective pilot evaluating clinician-rated accuracy/acceptability and the feasibility and patient-rated acceptability of integrating clinician-reviewed handouts into routine genitourinary (GU) oncology care.

## Methods

### Tool development

OncoEducate generates two-page, plain-language patient education handouts, designed to supplement clinician counseling, using a locked prompt and predefined content structure. The application uses the Claude Opus 4.1 LLM (Anthropic) with default generation settings (temperature = 1.0) and without parameter tuning or model fine-tuning. No patient-identifying information was entered into the model; clinicians input only diagnosis, regimen, and intent. The model was instructed to use plain-language and a patient-friendly tone, synthesize multi-drug regimens into cohesive explanations, and apply standardized phrasing for key concepts including treatment intent (Supplemental Material S1). The prompt included constraints such as avoiding overstating benefits and requiring an urgent-symptom section with specific thresholds (eg, fever > 100.4 °F). The prompt specified a preference for NCCN, ASCO, and NIH/NCI sources, but retrieval augmentation was not used. All handouts used a standardized seven-section structure (example in Supplemental Material S2).

### Study design

We conducted a two-phase pilot at a single academic cancer center, approved by the Penn Medicine AI Governance Committee and deemed exempt by the IRB. In Phase I, GU oncology clinicians (all study investigators) evaluated AI-generated handouts for nine commonly used palliative-intent regimens. Each clinician evaluated three handouts pertinent to their expertise, with each handout reviewed by two clinicians. Accuracy and acceptability were assessed using 7-point Likert-scale items. Feedback informed prompt refinement, including revised language for treatment intent and cancer staging, inclusion of drug brand names, grouping side effects by treatment category, and flexible dosing instructions. The same clinicians then reviewed and approved v2 handouts prior to patient-facing deployment. In Phase II, patients with advanced GU cancers initiating palliative-intent therapy received printed and electronic copies of the pre-approved handouts corresponding to their regimen. Eligible patients were ≥18 years old, English-speaking, and able to complete surveys. Socioeconomic status was characterized using the Area Deprivation Index (ADI) national ranking (1-100, higher=more disadvantaged, Neighborhood Atlas).^8^^,^^9^ Patients completed surveys at their next office or infusion visit or by telephone, at least one day after handout delivery.

### Outcomes and analysis

Primary outcomes were feasibility (proportion enrolled) and patient acceptability (7-point Likert scale items for informativeness, readability, and comfort with AI-assisted education). Secondary outcomes included clinician-rated accuracy and acceptability in Phase I. Exploratory outcomes included patient-reported perceived understanding and identification of palliative treatment intent. Identification of palliative treatment intent was measured using a validated 5-point response item, defining correct identification as selecting “not at all likely” when asked the likelihood of cure.^3^

## Results

### Clinician evaluation

**Figure 1.**
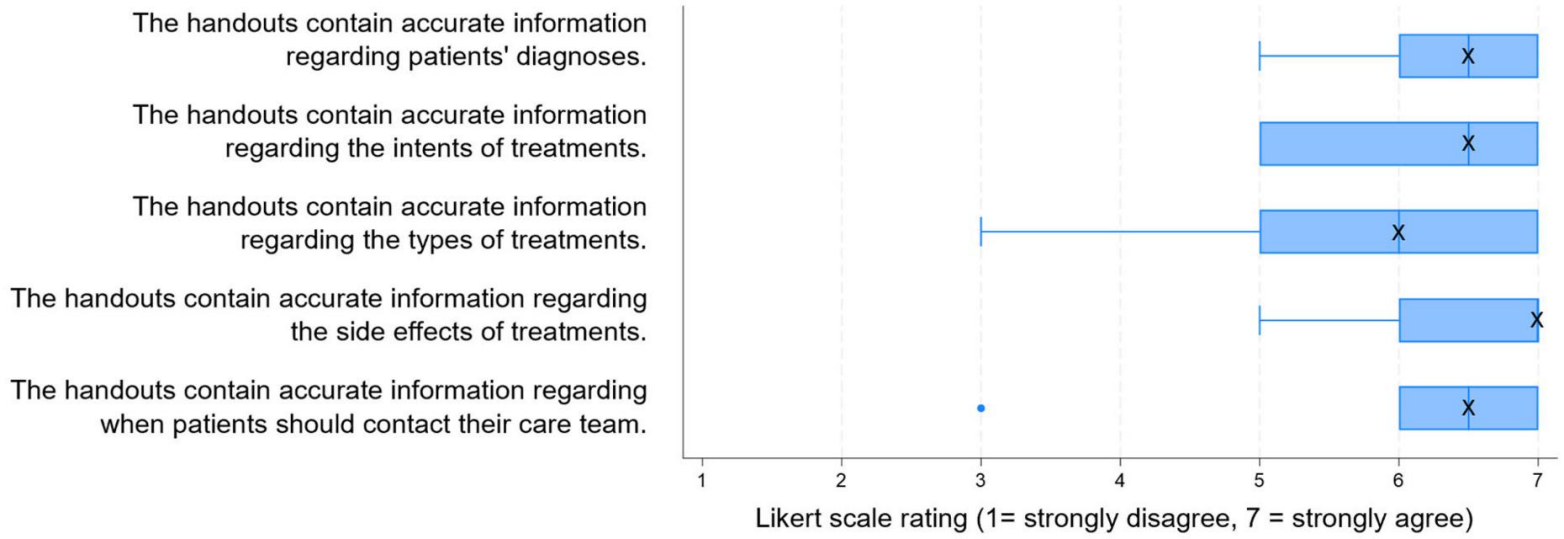
Oncology clinician accuracy ratings of OncoEducate Phase I handouts (*n* = 6). Box-and-whisker plots show oncology clinician ratings of the accuracy of OncoEducate Phase I content across five domains (7-point Likert scale; 1 = strongly disagree, 7 = strongly agree).

Five GU medical oncologists and one advanced practice provider (Supplemental Table S1) rated Phase I handouts as useful (median = 6/7, range: 5-7) and accessible to patients (median = 6.5/7, range: 5-7). Across five domains of accuracy, median accuracy ratings ranged from 6-7/7 (Figure 1). Clinicians expressed strong comfort with distributing AI-generated handouts (median = 7/7, range: 6-7) and with AI supplementing patient education (median = 7/7, range: 5-7).

### Patient evaluation

Over 8 weeks, 21 patients with advanced kidney, prostate, or urothelial cancers were approached; 20 enrolled (95%), with one patient declining. All 20 completed follow-up surveys. Median time from handout delivery to survey completion was 6 days (interquartile range [IQR]: 4.75-11.75). Most were male (85%) and White (85%). Urothelial (55%) and kidney (30%) cancers were most common. Median age was 70.5 years (IQR: 63.25-74.5). The median ADI national rank was 25 (IQR: 13-44). Enfortumab vedotin plus pembrolizumab was the most frequent regimen (40%), followed by ipilimumab plus nivolumab (15%).

**Figure 2.**
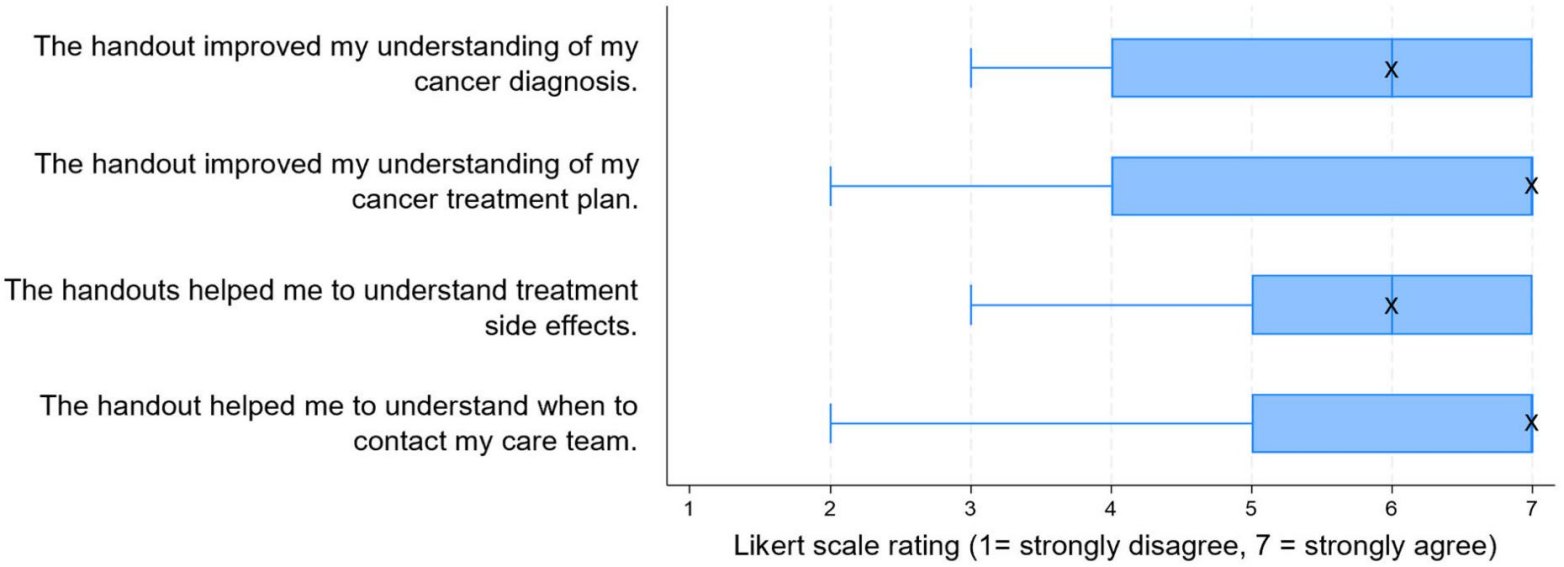
Patient-reported perceived understanding after OncoEducate Phase II handouts (*n* = 20). Box-and-whisker plots show patient ratings of perceived improvement in understanding after receiving OncoEducate Phase II handouts across four domains (7-point Likert scale; 1 = strongly disagree, 7 = strongly agree).

Patients strongly agreed that the handouts were informative (median = 7/7, IQR: 6.75-7) and readable (median = 7/7, IQR: 6.75-7). They reported strong perceived understanding of their treatment plan (median = 7/7, IQR: 4-7) and when to contact the care team (median = 7/7, IQR: 5.5-7) (Figure 2). Few patients reported prior use of AI for healthcare learning (median = 1/7, IQR: 1-2), but most were comfortable with AI-assisted patient education (median = 7/7, IQR: 5-7). Most patients (65%) correctly identified their treatment intent as palliative (Supplemental Figure S1).

## Discussion

OncoEducate achieved high accuracy, acceptability, and feasibility, and the 95% participation rate supports integrating clinician-reviewed AI-generated handouts into routine oncology care.

OncoEducate’s locked prompt and predefined template may reduce output variability compared with ad hoc use of general-purpose LLMs, while synthesizing multi-drug regimens into standardized explanations that consistently emphasize treatment intent. Because handouts were pre-approved by clinical scenario and reused, clinician oversight was maintained without per-encounter review burden. The primary advantage of generative AI in this setting is scalability: manually authoring handouts across the full landscape of regimen-diagnosis-intent combinations would be resource-intensive, whereas AI-generated content can be produced with consistent structure, reading level, and treatment-intent language.

The 65% rate of correct palliative-intent identification compares to 19%-31% in Weeks et al.^3^ Though causal inference cannot be drawn from this exploratory finding, these results suggest that structured, plain-language education may support recognition of palliative intent. This single item captures recognition of palliative intent rather than comprehensive prognostic understanding, and misidentification among 35% may reflect the complexity of processing intent information during treatment initiation.

This single-institution pilot has limited generalizability due to its small sample size, lack of a control arm, exclusion of non-English speakers, and predominantly White, male cohort. Sociodemographic characterization was limited to ADI, which captures educational attainment and correlates with area-level health literacy, but literacy level and handout readability were not formally assessed.^8^^,^^10^ Treatment-intent identification relied on a single validated item without baseline measurement. Content generation did not involve retrieval augmentation and verification was performed by clinician-investigators, introducing potential expectancy bias. These findings support future randomized trials in diverse cohorts with retrieval augmentation and efficacy outcomes.

## Supplementary Material

oyag135_Supplementary_Data

## Data Availability

The data underlying this article are available from the corresponding author upon reasonable request.
