# Supplementary material for: OncoEducate: a pilot study of generative AI to enhance patient–clinician communication in genitourinary cancer care
Source: Oncologist. 2026 Apr 9;31(5):oyag135. doi: 10.1093/oncolo/oyag135 (PMC13102169; doi:10.1093/oncolo/oyag135)
Supplement: oyag135_Supplementary_Data [file oyag135_supplementary_data.zip › Supplemental Table.docx]

**Supplemental Table S1: Characteristics of participating clinical reviewers (n=6)**

| **Clinician** | **Role** | **Years in Practice** | **Prior AI experience**  **(none/minimal/regular use)** |
| --- | --- | --- | --- |
| 1 | Medical oncologist | 25 | Minimal |
| 2 | Medical oncologist | 13 | Minimal |
| 3 | Medical oncologist | 10 | None |
| 4 | Medical oncologist | 6 | Minimal |
| 5 | Medical oncologist | 6 | Minimal |
| 6 | Advanced practice provider | 10 | None |
