# Supplementary material for: OncoEducate: a pilot study of generative AI to enhance patient–clinician communication in genitourinary cancer care
Source: Oncologist. 2026 Apr 9;31(5):oyag135. doi: 10.1093/oncolo/oyag135 (PMC13102169; doi:10.1093/oncolo/oyag135)
Supplement: oyag135_Supplementary_Data [file oyag135_supplementary_data.zip › Supplemental Dataset.pdf]

Supplemental Material S1: Prompt used to generate OncoEducate Version 2 handouts

You are tasked with creating a structured treatment handout for a cancer patient. This handout should be clear, informative, and easy for patients to understand. Follow these instructions to create the handout.

FORMATTING REQUIREMENTS

- Put the diagnosis on the same line as the word “Diagnosis” (e.g., “Diagnosis: Pancreatic cancer, stage IV”).
- Make ALL section headers bold and clearly visible.
- Use these exact section headers in bold: Diagnosis:, Treatment Intent:, Treatment Name:, About Your Treatment:, How Treatment is Given:, Common Side Effects:, Contact Your Care Team If You Experience:

Begin by creating a handout with the following sections:

Diagnosis: ${cancerType}

Treatment Intent: ${treatmentIntent}

If curative is selected, include the following explanation:
Your doctors believe there's a good chance treatment can remove or destroy all the cancer. The goal is for the cancer to go away and not come back.

If palliative is selected, include the following explanation:
This treatment is not meant to cure the cancer. The cancer is at an advanced stage and unlikely to completely disappear. The goal is to help you feel better and live longer. Treatment can slow down or shrink the cancer and help manage symptoms.

Treatment Name: ${treatmentName || "Not specified"}

About Your Treatment:

- A brief explanation of the treatment.
- If there are similar treatments that could be used for this diagnosis and stage and this particular treatment has unique advantages, briefly describe the advantage without overstating or overpromising benefits.
- Use bullet points for all information in this section.

How Treatment is Given:

- Use bullet points for all content in this section.
- For each drug, start with its name and explain how and when it is given.

- Use plain language and avoid medical abbreviations (e.g., say "through a vein (IV)" instead of "IV push").
- Use the "Day X" format when referencing specific days (e.g., "Day 1," "Day 8").
- If medications are given together, clearly state this.
- If they are given on different days or cycles, explain when and how.
- If a drug is taken by mouth, include how often it's taken (e.g., daily), when (e.g., with or without food), and any other relevant instructions.
- When describing dosing schedules, always reflect common variation in clinical practice. Many cancer drugs have more than one commonly used schedule. Present the most typical schedule first, then mention alternative options.

Example:
Pembrolizumab (Keytruda): Given through a vein (IV) on Day 1, usually every 3 weeks, though some patients receive it every 6 weeks depending on their doctor's recommendation.

Acknowledge that schedules may vary based on the patient's health, doctor's judgment, or updated treatment guidelines. Add a sentence like: "Your doctor may adjust the schedule based on side effects, lab results, or other factors."

If any monitoring is required—like labs, EKGs, or imaging—mention this briefly and clearly.

Common Side Effects:

- List 5–10 important side effects.
- Organize by treatment category with generic medication names in parentheses:
  • Chemotherapy Side Effects (generic names of chemo drugs in this treatment):
  • Targeted Therapy Side Effects (generic names of targeted therapy drugs in this treatment):
  • Hormone Therapy Side Effects (generic names of hormone therapy drugs in this treatment):
  • Immunotherapy Side Effects (generic names of immunotherapy drugs in this treatment):

If immunotherapy side effects are listed, use the following explanation, with each sentence as its own bullet point and no additional text:

- Mild symptoms like fatigue, rash, or joint pain are common.
- Immunotherapy can cause inflammation in normal organs, which may be serious.
- If two or more immunotherapy drugs are used in this treatment, include this additional bullet point: "The risk of side effects is higher when two immunotherapy drugs are used together."

- Treat any new or worsening symptom—such as diarrhea, cough, fatigue, shortness of breath, or headache—as possibly related to treatment.
- Some side effects may require holding treatment or starting steroids.

Contact Your Care Team If You Experience:

- Create a list of 5–10 urgent symptoms or scenarios that clearly warrant contacting the care team.
- Use plain language and include specific thresholds (e.g., “Fever over 100.4°F”).
- Keep the tone calm, direct, and patient-friendly.
- Always include the following:
  • Fever over 100.4°F (38°C)
  • New or worsening shortness of breath
  • Chest pain or pressure
  • Uncontrolled nausea, vomiting, or diarrhea
- In addition to these core symptoms, include 2–5 urgent additional symptoms that reflect risks specific to the treatment regimen.
- Avoid vague statements such as “any new symptoms.”
- If one of the treatment medications is to be given through a pump at home (such as 5-FU), include as a reason to call if pump malfunctions.

Write the handout in a clear, compassionate, and patient-friendly tone. Avoid using complex medical jargon, and when technical terms are necessary, provide simple explanations. Remember to tailor the information to the specific cancer type and treatment provided in the input variables.

IMPORTANT:

- Anytime a generic drug name is mentioned, include commonly prescribed U.S. brand name(s) in parentheses. Example: abiraterone (Zytiga).
- Avoid using “Stage IV” or “Stage 4” in the handout title. If either term is used, replace it with “Advanced.”
- Base your explanation on trusted sources such as NCCN guidelines, ASCO, and/or NIH/NCI patient materials.

Supplemental Material S2: Example Phase II Handout for Enfortumab Vedotin plus Pembrolizumab

Diagnosis: Advanced bladder cancer

Treatment Intent: Palliative

This treatment is not meant to cure the cancer. The cancer is at an advanced stage and unlikely to completely disappear. The goal is to help you feel better and live longer. Treatment can slow down or shrink the cancer and help manage symptoms.

Treatment Name: Enfortumab vedotin (Padcev) + pembrolizumab (Keytruda)

About Your Treatment:

• This combination uses two different types of cancer medicines that work in different ways

• Enfortumab vedotin (Padcev) is a targeted therapy that delivers cancer-killing medicine directly to bladder cancer cells

• Pembrolizumab (Keytruda) is immunotherapy that helps your immune system recognize and fight cancer

• This combination has shown better results than chemotherapy alone for advanced bladder cancer

• Treatment continues as long as it's working and side effects are manageable

How Treatment is Given:

• Both medicines are given through a vein (IV) in the same visit

• Enfortumab vedotin (Padcev): Given through a vein on Days 1 and 8 of each 21-day cycle

• Pembrolizumab (Keytruda): Given through a vein on Day 1, usually every 3 weeks, though some patients receive it every 6 weeks depending on their doctor's recommendation

• Your doctor may adjust the schedule based on side effects, lab results, or other factors

• Blood tests are needed before each treatment to check blood counts and organ function

• Periodic scans will check how treatment is working

Common Side Effects:

Targeted Therapy Side Effects (enfortumab vedotin):

• Skin reactions including rash, dry skin, or itching

• Nerve problems causing numbness or tingling in hands and feet

• Hair loss

• Decreased appetite and weight loss

• High blood sugar levels

Immunotherapy Side Effects (pembrolizumab):

• Mild symptoms like fatigue, rash, or joint pain are common

• Immunotherapy can cause inflammation in normal organs, which may be serious

• Treat any new or worsening symptom—such as diarrhea, cough, fatigue, shortness of breath, or headache—as possibly related to treatment

• Some side effects may require holding treatment or starting steroids

Contact Your Care Team If You Experience:

• Fever over 100.4°F (38°C)

• New or worsening shortness of breath

• Chest pain or pressure

• Uncontrolled nausea, vomiting or diarrhea

• Severe skin rash, blistering, or peeling skin

• Numbness, tingling, or burning sensation in hands or feet that interferes with daily activities

• Signs of high blood sugar: excessive thirst, frequent urination, blurred vision

• Eye pain, vision changes, or light sensitivity

• Severe fatigue that prevents you from getting out of bed
