# Supplementary material for: OncoEducate: a pilot study of generative AI to enhance patient–clinician communication in genitourinary cancer care
Source: Oncologist. 2026 Apr 9;31(5):oyag135. doi: 10.1093/oncolo/oyag135 (PMC13102169; doi:10.1093/oncolo/oyag135)
Supplement: oyag135_Supplementary_Data [file oyag135_supplementary_data.zip › Supplemental Figure.pdf]

Supplemental Figure S1: Patient responses to treatment-intent identification question (n=20)

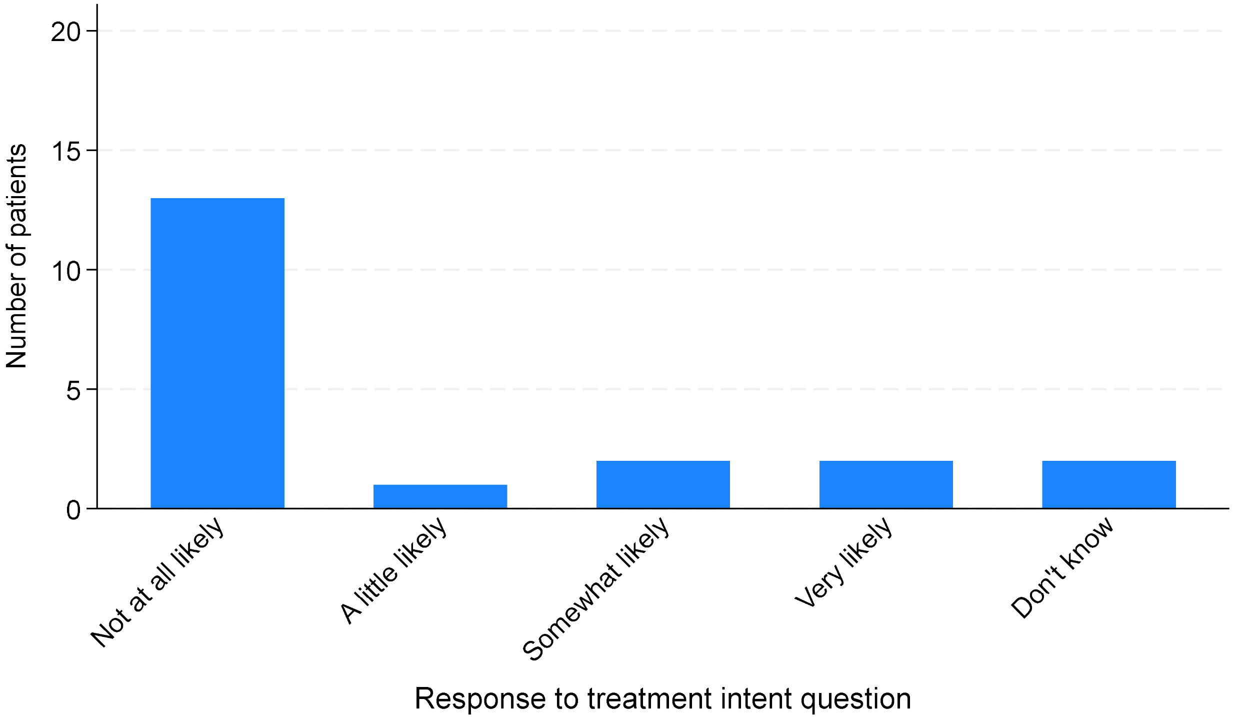

Bar chart showing the distribution of patient responses to the multiple-choice question "How likely is the treatment to cure your cancer?"
